# Biological determinants of physical activity across the life course: a “Determinants of Diet and Physical Activity” (DEDIPAC) umbrella systematic literature review

**DOI:** 10.1186/s40798-018-0173-9

**Published:** 2019-01-08

**Authors:** Katina Aleksovska, Anna Puggina, Luca Giraldi, Christoph Buck, Con Burns, Greet Cardon, Angela Carlin, Simon Chantal, Donatella Ciarapica, Marco Colotto, Giancarlo Condello, Tara Coppinger, Cristina Cortis, Sara D’Haese, Marieke De Craemer, Andrea Di Blasio, Sylvia Hansen, Licia Iacoviello, Johann Issartel, Pascal Izzicupo, Lina Jaeschke, Martina Kanning, Aileen Kennedy, Fiona Ling, Agnes Luzak, Giorgio Napolitano, Julie-Anne Nazare, Camille Perchoux, Tobias Pischon, Angela Polito, Alessandra Sannella, Holger Schulz, Rhoda Sohun, Astrid Steinbrecher, Wolfgang Schlicht, Walter Ricciardi, Ciaran MacDonncha, Laura Capranica, Stefania Boccia

**Affiliations:** 1 Università Cattolica del Sacro Cuore, Sezione di Igiene, Istituto di Sanità Pubblica, Rome, Italy; 2 Leibniz Institute for Prevention Research and Epidemiology-BIPS, Bremen, Germany; 3 Department of Sport, Leisure and Childhood Studies, Cork Institute of Technology, Cork, Munster Ireland; 4 Department of Movement and Sports Sciences, Ghent University, Ghent, Belgium; 5 Department of Physical Education and Sport Sciences, University of Limerick, Limerick, Ireland; 6 Department of Applied Sciences in Physical Activity and Management, Catholic University of Valencia “San Vicente Mártir”, Valencia, Spain; 7 Council for Agricultural Research and Economics, Research Centre for Food and Nutrition, Rome, Italy; 8 Department of Movement, Human and Health Sciences, University of Rome Foro Italico, Rome, Italy; 9 Department of Human Sciences, Society, and Health, University of Cassino and Lazio Meridionale, Cassino, Italy; 10 Department of Medicine and Aging Sciences, G. d’Annunzio’ University of Chieti-Pescara, Chieti and Pescara, Italy; 11 Department of Sport and Exercise Sciences, University of Stuttgart, Stuttgart, Germany; 12 Department of Epidemiology and Prevention, IRCCS Istituto Neurologico Mediterraneo: NEUROMED, Pozzilli, Italy; 13 School of Health and Human Performance, Multisensory Motor Learning Lab, Dublin City University, Dublin, Ireland; 14 Molecular Epidemiology Group, Max Delbruck Center for Molecular Medicine in the Helmholtz Association (MDC), Berlin, Germany; 15 Centre for Preventive Medicine, School of Health and Human Performance, Dublin City University, Dublin, Ireland; 16 Institute of Sport, Exercise & Active Living, Victoria University, Melbourne, Australia; 17 Institute of Epidemiology I, Helmholtz Zentrum München, German Research Center for Environmental Health, Neuherberg, Germany; 18 Centre de Recherche en Nutrition Humaine Rhône-Alpes, CarMeN INSERM U1060, University of Lyon1, Lyon, France; 19 Italian National Institute of Health, (Istituto Superiore di Sanita - ISS), Rome, Italy; 20 Fondazione Policlinico Universitario A.Gemelli IRCCS, UOC Igiene Ospedaliera, Roma, Italia

**Keywords:** Physical activity, Biological determinants, Umbrella systematic review

## Abstract

**Background:**

Despite the large number of studies and reviews available, the evidence regarding the biological determinants of physical activity (PA) is inconclusive. In this umbrella review, we summarized the current evidence on the biological determinants of PA across the life course, by pooling the results of the available systematic literature reviews (SLRs) and meta-analyses (MAs).

**Methods:**

We conducted an online search on MEDLINE, ISI Web of Science, Scopus, and SPORTDiscus databases up to January 2018. SLRs and MAs of observational studies that investigated the association between biological determinants of PA and having PA as outcome were considered eligible. The extracted data were assessed based on the importance of the determinants, the strength of evidence, and the methodological quality.

**Results:**

We identified 19 reviews of which most were of moderate methodological quality. Determinants that were studied most frequently among all ages and demonstrated evidence suggesting a positive association to PA were younger age, being male, higher health status, and higher physical fitness levels. Among adults, normal birth weight was found to be positively associated to PA with convincing strength of evidence, while findings among adolescents were inconsistent and with limited strength of evidence.

**Conclusions:**

Different social or behavioral factors may contribute to the decrease of PA with age and among females versus males, and creating programmes targeted at diverse ages, female population, and adults with abnormal birth weight is recommended. Future studies should use prospective study designs, standardized definitions of PA, and objective measurement methods of PA assessment.

## Key Points

- Younger age, being male, higher health status and higher physical fitness levels suggested a positive association with physical activity.
- Normal birth weight was positively associated with physical activity among adults.
- Different social and behavioral factors contribute to the decrease of physical activity with increasing age.

## Background

The World Health Organization (WHO) has developed global recommendations to increase the amount of physical activity (PA) in the general population, following the abundant evidence of the positive effects of PA on the maintenance of cardiovascular health and metabolic index, thus being of high importance for the prevention and the management of the non-communicable diseases (NCDs) [1]. Since NCDs constitute a large part of the worldwide disease burden, prevention programs with the effective incorporation of PA are of paramount importance [1–3].

Biological determinants can be all the individual characteristics of a person that have biological background, including genetics, family predisposition, pathology, health status, anthropometry, body mass index (BMI)/adiposity, birth weight, physical fitness levels, age, sex, ethnicity, etc. [4]. Even though some of them are non-modifiable, they influence the patterns of PA interacting with other factors on multiple levels [5–8]. Because of that, they should be considered when investigating PA participation and introducing new interventions of PA.

Several original studies, systematic literature reviews (SLRs), and meta-analyses (MAs) evaluating the determinants promoting or inhibiting PA participation are available in the literature. Specifically concerning biological determinants of PA, a number of primary epidemiological studies, SLRs and MAs, and two umbrella reviews [9, 10], the last concerning only young populations, have been published. According to all these studies, lower age and being male were generally found to be positively associated with PA in most of them and there is inconsistent evidence for the association between PA and several additional biological determinants (e.g., BMI, ethnicity, health status, and family risk). Among studies, there is wide variability of study aims and measurement methods and classifications used in assessing PA. This produces variability of study results and as a result, a lack of precise evidence about the biological determinants of PA participation. Furthermore, in order to establish experimental evidence related to PA, a clear understanding of associations or predictive relationships between determinants is needed [11].

Hence, the aim of this umbrella systematic review is to give an overview of the studies investigating biological determinants influencing PA across the life course by systematically reviewing the available evidence from existing SLRs and MAs (referred as “reviews” in the text) of primary observational studies. As PA is beneficial for health of people of any age, we did not restrict the overview to a particular age group. Additionally, we assessed the overall results of the retrieved reviews in terms of the importance of the determinant, the strength of the evidence, and the methodological quality of the reviews.

## Methods

This umbrella review is part of the “Determinants of Diet and Physical Activity” (DEDIPAC) project (https://www.dedipac.eu/), which was planned to include seven umbrella reviews on determinants of PA (biological, psychological, behavioral, physical, socio-cultural, economic, and policy). The current umbrella review focuses solely on the biological determinants of PA.

We drafted this manuscript following the PRISMA checklist [12]. The protocol of the umbrella systematic review is registered on PROSPERO (Record ID: *CRD42015010616*), the international prospective register of systematic reviews [13].

### Search Strategy and Eligibility Criteria

We used the same search strategy for all the seven umbrella reviews, extracting at the end only the articles that included biological determinants. We systematically searched electronic databases for SLRs and MAs investigating the determinants of PA across the life course. An online search was conducted on the following search engines: MEDLINE, ISI Web of Science, Scopus, and SPORTDiscus. The search was limited to reviews published in English language from January 2004 to January 2018. In order to summarize the current knowledge on determinants of PA, we did not include the reviews published before 2004. Table 1 shows the MEDLINE search strategy; this was also used as the template for the search strategies in the other databases.

**Table 1:** Search strategy: key words used for the literature research

Set	Search terms
#1	“physical activit*” OR “physical exercise*” OR sport OR “motor activit*” OR “locomotor activit*” OR athletic* OR fitness OR “physical movement*” OR “physical performance*” OR “aerobic exercise*” OR “physical effort*” OR “physical exertion*”
#2	determinant OR determinants OR correlator OR correlators OR mediator OR mediators OR moderator OR moderators OR contributor OR contributors OR factor OR factors OR association OR modifier OR modifiers OR confounder OR confounders OR pattern OR patterns OR predictor*
#3	demographic* OR motivation OR cognition OR emotion* OR attitude* OR “self-perception” OR “self-confidence” OR “self-efficacy” OR competence OR reward* OR success* OR challenge* OR knowledge OR belief* OR “personal trait*” OR “body image” OR satisfaction OR “time availability” OR “perceived environment” OR family OR peer* OR school* OR leader* OR coach* OR group* OR “climate” OR network* OR employment OR retirement OR “educational level” OR SES OR “socioeconomic status” OR “local identity” OR “national identity” OR value* OR tradition* OR “social expectation*” OR “social trend*” OR “social barriere*” OR “availability of tool*” OR “availability of service*” OR “access to tool*” OR “access to service*” OR neighborhood OR “community route*” OR “school environment” OR “work environment” OR architecture OR urbanization OR transport OR traffic OR “facilit* in public space*” OR advertisement OR “availability of sport club*” OR “availability of fitness center*” OR advocacy OR lobbying OR “corporate social responsibility” OR “physical activity promotion initiative*” OR legislation OR health OR education OR tourism OR environment OR “urban planning” OR transport* OR sport OR sports OR culture OR dance OR theater OR “gender mainstreaming” OR “social inclusion” OR “fiscal measure*” OR program* OR plan OR plans OR communication OR media OR guideline*
#4	“systematic literature review” OR “meta-analysis”

SLRs and MAs of observational primary studies, done on participants at any age, on the association between any determinant and PA, or exercise, or sport as main outcome, were included in the umbrella review. The following were excluded: (i) SLRs and MAs of intervention studies; (ii) SLRs and MAs that did not focus on the general population (e.g., reviews of studies done on patients, athletes, specific professions); and (iii) umbrella systematic reviews on the same topic (e.g., reviews of SLRs or MAs of epidemiological studies on determinants associated with PA).

### Selection Process

Across all databases, our search identified a total number of 18,516 potentially relevant papers. After the removal of duplicates, 15,147 papers remained. Relevant papers were independently screened and assessed by two reviewers belonging to the DEDIPAC KH (Knowledge Hub), who screened the titles and if necessary, the abstracts, and the full texts. Before the final study inclusion or exclusion, a common decision was reached for each study. Any uncertainty and disagreement was resolved by consulting three further authors (SB, LC, AP).

**Fig. 1:**
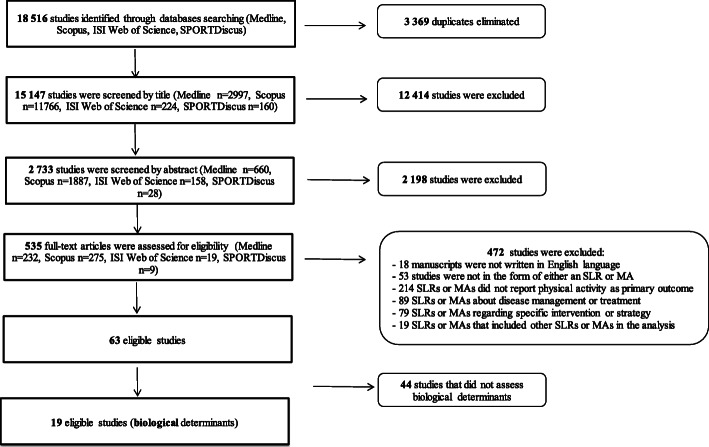
Flowchart of the literature research by database. *MA* meta-analysis, *SLR* systematic literature review

As summarized in Fig. 1, after title and abstract reading, 12,414 and 2198 articles were respectively excluded because they did not meet the inclusion criteria. Thus, a total number of 535 full-text articles were assessed for eligibility, which resulted in inclusion of 63 eligible papers. Of these, 44 reviews did not concern biological determinants of PA. Therefore, the final number of reviews included in the present umbrella review on biological determinants of PA was 19.

### Data Extraction

For each included review, we extracted data on predefined extraction forms, developed by the two authors (KA, AP) and verified by the DEDIPAC KH, which include the following information: year of publication, type of review (SLR or MA), number of eligible primary studies included over the total number of studies included in each review; continent/s of the included studies, primary study design, overall sample size, age range or mean age, sex proportion, year of publication range of included studies, outcome details, type of determinant/s, aim of the review; overall results (qualitative or quantitative), overall recommendations, and limitations as provided by the review itself.

### Evaluation of Importance of Determinants and Strength of the Evidence

We summarized the retrieved results from the eligible studies combining two grading scales, used previously by Sleddens et al. [14]. One of the scales grades the importance of the determinants (referring to the consistency of the associations among reviews/individual studies) and the other grades the strength of evidence (referring to the study design used among individual studies).

According to the scale for the importance, a determinant can score a (−−) if all reviews, without exception report no association between the determinant and the outcome, a (−) if the association was found in less than 25% of the reviews or of the original studies, and a (0) if the results are mixed, or more specifically, that the variable has been found to be a determinant and/or reported a (non)-significant effect size larger than 0.30 in 25% to 75% of the available reviews or of the primary studies analyzed in these reviews. Furthermore, the importance of the determinant scores a (+) if the association was found in more than 75% of the reviews or of the included individual studies and a (++) if association was found in all reviews, without exception.

The strength of the evidence is described as “convincing” (convincing evidence, Ce) if it is based on studies that show consistent associations and have longitudinal design with sufficient size and duration, whereas evidence of “probable” association (probable evidence, Pe) can be given to determinants showing fairly consistent associations based upon at least two cohort studies. In the second case, there are some shortcomings either in terms of the consistency of the results or other aspects such as limited duration of the studies, small sample sizes, or inadequate follow up. Furthermore, “limited suggestive evidence” (Ls) is given to determinants for which there is insufficient number of longitudinal studies and “limited, no conclusive evidence” (Lns) when the evidence for the associations between a determinant and the outcome are based solely on studies of cross-sectional design [14].

### Quality Assessment

We assessed the methodological quality of the included reviews using a modified version of the AMSTAR Checklist [15]. The question number 11 referring to the presence of any conflict of interest was modified after a consensus between the DEDIPAC KH partners, so that the conflict of interest was evaluated in the reviews included and not in the primary studies included in each review.

The included SLRs and MAs were independently evaluated by two reviewers belonging to the DEDIPAC KH. Any uncertainty and disagreement was resolved by consulting three further authors (SB, LC, AP). The eleven criteria were evaluated and scored as a 1 when the criterion was applicable to the analyzed review or as a 0 when the criterion was not applicable to the analyzed review. As a consequence, the total quality score for each included review ranged from 0 to 11. The quality of the review was labeled as weak (score ranging from 0 to 3), moderate (score ranging from 4 to 7), or strong (score ranging from 8 to 11).

## Results

### Characteristics of the SLRs and MAs Included

The characteristics of the 19 included SLRs and MAs (14 and 5 respectively) are summarized in Table 2. Since some of them included primary studies that examined the associations between non-biological determinants and PA, we did not appraise all the primary studies included in the individual SLRs or MAs in our umbrella review.

**Table 2:** Characteristics of the included studies

Study/type of review	Number of individual studies included in the umbrella review/total number of studies included in the review	Continent/s	Study design	Sum of the size of the individual samples included (sample size range)	Age range or mean (years)	Female sex %	Year publication (range)
Barnett et al. (MA) [34]	50/59	Europe (*n* = 16), North America (*n* = 16), South America (*n* = 2), Asia (*n* = 5), Africa (*n* = 1), Australia (*n* = 5)	Cohort (*n* = 6) Cross-sectional (*n* = 44)	36,196 (34–7175)	3.6–14.5	N.A.	1997–2014
Oglund et al. (MA) [31]	11/11	Europe (*n* = 6), North America (*n* = 1), South America (*n* = 3), Asia (*n* = 1), Australia (*n* = 1)	Cohort (*n* = 11)	26,384 (44–7736)	0–15 (10.2)	48–56	2006–2013
Olsen et al. (SLR) [22]	13/21	N.A.	Cross-sectional (*n* = 7) Cohort (*n* = 1) Case-control (*n* = 5)	9012 (17–1877)	19–(≥ 65)	100	2000–2010
Babakus et al. (SLR) [24]	38/38	Europe (*n* = 22), North America (*n* = 10), New Zealand (*n* = 1), Australia/Asia (*n* = 2), South America (*n* = 1), Asia (*n* = 3)	Cohort (*n* = 1) Cross-sectional (*n* = 37)	552,967 (56–347,229)	N.A.	40–100	1980–1912
Barnett et al. (SLR) [33]	5/5	Europe (*n* = 3), North America (*n* = 2)	Cross-sectional (*n* = 5)	N.A.	N.A.	N.A.	1980–2010
De Craemer et al. (SLR) [18]	22/43	North America, Europe, Australia, New Zealand	Cohort (*n* = 6) Cross-sectional (*n* = 35) Cross-sectional and cohort (*n* = 1)	N.A.	4–6	N.A.	1990–2010
Ridgers et al. (SLR) [17]	47/53	Multiple continents	Cross-sectional (*n* = 42) N.A. (*n* = 5)	N.A.	5–18	N.A.	1990–2011
Stanley et al. (SLR) [19]	12/22	Europe (*n* = 4) Australia (*n* = 4) North America (*n* = 4)	Cross-sectional (*n* = 12)	N.A.	8–14	N.A.	1990–2011
Uijtdewillingen et al. (SLR) [28]	25/30	North America (*n* = 14), Europe (*n* = 9), New Zealand (*n* = 1), Asia (*n* = 1)	Cohort (*n* = 25)	41,244 (22–12,812)	3–17 Children = 4–12 Adolescents = 13–18	40–100	2005–2010
Craggs et al. (SLR) [29]	30/46	North America (*n* = 20), Europe (*n* = 9), Australia (*n* = 1)	Cohort (*n* = 30)	59,323	4–9 (*n* = 3) 14–18 (*n* = 8) 10–13 (*n* = 19)	50–100	Up to 2010
Dumith et al. (MA) [30]	26/26	North America (*n* = 17), Europe (*n* = 8), New Zealand (*n* = 1)	Cohort (*n* = 26)	43,341 (97–1279)	10–19	55	1977–2007
Koeneman et al. (SLR) [32]	12/30	North America (*n* = 6), Europe (*n* = 3), Australia (*n* = 1), Asia (*n* = 2)	Cohort (*n* = 12)	N.A.	> 40	N.A.	1992–2010
Siddiqi et al. (SLR) [27]	21/29	N.A.	Cross-sectional (*n* = 21)	878 (15–89)	≥ 18	45–100	1995–2009
Andersen et al. (MA) [16]	13/13	Europe (*n* = 13)	Cohort (*n* = 13)	43,482 (225–4363)	14–74	43	1970–2008
Hinkley et al. (SLR) [21]	20/24	North America (*n* = 13), Europe (*n* = 7)	Cohort (*n* = 3) Cross-sectional (*n* = 17)	8469 (30–3141)	43,222	45–55	1980–2006
Tzormpatzakis et al. (SLR) [23]	36	N.A.	Cross-sectional (*n* = 36)	74,280	≥ 15	0–56	1993–2006
Van der Horst et al. (SLR) [20]	30/60	N.A.	Cross-sectional (*n* = 25) Cohort (*n* = 5)	N.A.	4–12 (*n* = 9) 13–18 (*n* = 22)	N.A.	1999–2005
Coble et al. (SLR) [25]	22/35	N.A.	Cross-sectional (*n* = 22)	29,623 (30–4549)	≥ 18	50–100	1991–2005
Rhodes et al. (MA) [26]	15/35	North America (*n* = 6), Europe (*n* = 8), Asia (*n* = 1)	Cross-sectional (*n* = 10) Cohort (*n* = 6)	51,537 (35–22,448)	19–77 (15–74)	0–100	1969–2006

*MA* meta-analysis, *SLR* systematic literature review, *N*.*A*. not available from the review; included studies are the original studies that assessed biological determinants

Most of the reviews included primary studies from multiple continents, mostly Europe (14 reviews), North America (13 reviews), and Australia (8 reviews). One review included cohort studies conducted only in Europe [16]. In 11 of the included reviews, most of the primary studies were cross-sectional [16–27], but there was also a considerable number of reviews that included prospective and cohort studies [16, 18, 20–22, 24, 26, 28–31]. In six reviews, it was not possible to retrieve the total population sample size of the included studies [17–20, 32, 33], and two reviews provided only the sum of the individual studies’ sample sizes [23, 29]. In the remaining studies, the total population sample size ranged from 878 to 522,967. Some reviews did not report the age of the participants in the primary studies [24, 33]. Finally, the percentage of the female participants, if reported, ranged from 0 to 100% of the total sample size, though these data were absent in some studies [17–20, 32–34] (Table 2).

### Investigated Determinants of the Reviews

Table 3 summarizes the findings of the included reviews on the associations between the biological determinants of PA. The most frequently studied determinants were age (*n* = 13) [17–23, 25, 26, 28, 29, 32, 34], sex (*n* = 14) [17–21, 23–26, 28, 29, 32–34], and ethnicity (*n* = 10) [17–21, 24, 28, 29, 32, 33]. BMI or overweight were assessed in nine reviews [17, 19–22, 25, 28, 32, 34]; two reviews included the family risk in their investigations [18, 21], five reviews examined the health status of the participants [21, 22, 25, 27, 32], and six reviews investigated physical fitness levels/motor function/motor skills/energy levels as determinants of PA [17–19, 21, 22, 27]. Furthermore, birth weight was studied in three reviews [16, 18, 31], and anthropometry or body shape/waist circumference in two [18, 29]. Finally, two reviews included maturation/level of development in adolescents [17, 29], one special educational needs as determinants of PA [17], and one included early growth and motor development [31].

**Table 3:** Results of the included reviews

Study/type of review	Outcome(s)	Determinant(s)	Review aim	Overall qualitative results of the review	Overall quantitative results of the review	Overall limitations of the study	Overall recommendations
Barnett et al. (MA) [34]	Object control movement skill, competency, locomotor skill competency, stability, motor coordination, skill composite	Age, sex, BMI	To identify factors correlated with motor competence	Age is positively correlated with physical activity, while adiposity is negatively. Boys are more skilled than girls for object control and motor coordination	Correlation coefficient for age: 0.37, 95% CI 0.29–0.45. 0.45, 95% CI 0.36–0.53. 0.34, 95% CI 0.29–0.39. Correlation coefficient for sex: 0.28, 95% CI 0.20–0.36	Few studies focused on the same correlate and the same motor skill outcome. Few studies provided correlation coefficients	Additional research that investigates the role of many correlates of motor competence
Oglund et al. (MA) [31]	Overall PA	Birth weight, motor development, early growth	To explore whether birth weight, early growth and motor development act as determinants of physical activity in children and youth	Birth weight is not an important determinant of physical activity in youth. Available data do not allow firm conclusions whether early growth and motor development act as determinants of physical activity in youth	b = −3.08, 95% CI − 10.20, 4.04	Several of the studies had limitations impacting the quality of the results, but these were not necessarily captured in the standardized quality assessment	More data from high quality birth-cohort studies are warranted before firm conclusions can be made
Olsen et al. (SLR) [22]	PA behaviour	Health, age, lack of energy, weight Health, age, lack of energy, weight	To identify factors that influence PA in rural women	Rural women were found to be less active and experience more barriers to PA than urban women; PA determinants among rural women can be categorized according to personal, socioeconomic, and physical environment factors	N.A.	Evaluation of data and analysis was done by one reviewer only; the terms “rural” and “PA” were inconsistently defined among studies; exclusion of articles studying women outside USA	Additional research that clearly defines and consistently applies the terms rural and PA is needed to strengthen knowledge in this area
Babakus et al. (SLR) [24]	Mixture of PA (total, leisure time, home, work, active commuting, energy expenditure, occupational, intensity, steps or physical inactivity) and sitting time	Sex, ethnicity	To assess what is known about the levels of PA and sedentary time and to contextualize these behaviors among South Asian women with an immigrant background	South Asian women were less active than the other ethnic groups as well as compared to South Asian males	N.A.	No standardized method for quality evaluation; lack of details from some of the included papers; publication and researcher bias possibility; significant heterogeneity among studies	More research should be dedicated to standardize objective PA measurement and to understand how to utilize the resources of the individuals and communities to increase PA levels and overall health of South Asian women
Barnett et al. (SLR) [33]	PA change across transition to retirement (secondary: leisure time PA, structured exercise, total PA)	Sex, ethnicity	To gain a deeper understanding of qualitative evidence on PA around the transition to retirement	Overall, exercise and leisure time PA increased after the transition to retirement, whereas the findings regarding changes in total PA were inconclusive; men tend to be more active than women	N.A.	Limited number of studies with population from limited socioeconomic diversity; different approaches to assess PA between studies	Future research should address predictors of maintenance of recreational PA after the transition to retirement, the broader benefits of PA, and barriers to PA among retirees from lower occupational groups
De Craemer et al. (SLR) [18]	Overall PA, MVPA, active transport, during recess	Sex, family risk, preterm birth, birth weight, age, ethnicity, waist circumference, movement skills	To systematically review the correlates of PA, sedentary and eating behaviour in pre-school children 4–6 years old	Little support for biological correlates and PA in general; strong correlation with sex and age and PA; negative association with family obesity risk and positive correlation with gestational age	N.A.	Some limitations regarding the coding of the association of the variables; several studies included wider age range	Strategies should target both boys and girls, all ethnic groups, and parents of both low and high SES; especially on weekdays, should be a focus on maintaining the level of PA and decreasing the level of sedentary behaviour; on weekends, the focus should be on increasing the level of PA
Ridgers et al. (SLR) [17]	Recess PA	Age, sex, BMI/overweight, body mass, maturation, ethnicity, fitness, special educational needs	To examine the correlates of children’s and adolescent’s PA during school recess periods	Boys are more physically active during recess, no association was found for BMI/central adiposity and grade level	N.A.	The majority are small-sized and cross-sectional studies; MA is difficult to obtain given the limited number of studies and the lack of consistency between them; lack of objective measures	More research is needed concerning correlates of PA in recess period, particularly in adolescents; schools to increase overall facility provision, unfıxed equipment and methods to increase social support, particularly by peers
Stanley et al. (SLR) [19]	School break time PA cross-sectional studies	Age, sex, motor skills, BMI, ethnicity	To identify the correlates of children’s PA (8–14 years) occurring during the school break time and after school periods	Boys and younger children tend to be more active during break time and after school; BMI in females negatively associated with after school PA, age was negatively associated in school break and after school	N.A.	Small number of studies that vary in methodological aspects; possibility that some studies are missed during the search process; majority of cross-sectional studies	Need for high quality evidence upon which PA promotion in young people can be tailored to specific settings and contexts
Uijtdewillingen et al. (SLR) [28]	Overall PA	Age, sex, ethnicity, BMI/skinfolds	To summarize and update the existing literature on determinants of PA and sedentary behavior in young people	Moderate evidence of positive relationship between age PA and negative relationship between ethnicity and PA among adolescents	N.A.	Included studies assessed overall PA only; used two databases only; the selected language of publication was English only	To develop long- term interventions more prospective studies with high quality are needed
Craggs et al. (SLR) [29]	Overall PA	Sex, anthropometry, ethnicity, age, developmental state,	To systematically review the published evidence regarding determinants of change in PA in children and adolescents	Inconclusive associations were reported for large proportion of the determinants examined	N.A.	Possibility of publication bias (included published studies only); heterogeneity in study samples, exposure and outcome measures included in this review; some studies draw data from the same cohorts; semi-quantitative reporting used in the review that limits the classification of the associations	Further research should include objective measures of PA and use previously validated questionnaires to assess the investigated determinants; more high quality research is needed in all age groups, especially in younger children
Dumith et al. (MA) [30]	Overall PA	Age	To systematically review the international literature regarding PA change in adolescence and quantify that change	The decline in PA during adolescence is consistent finding among studies. In the later studies the decline is more prominent among girls than in boys, although these differences are not significant.	Mean decline (95% CI) 7.0 (5.2–8.8)	Lack of methodological evaluation of the studies included; some studies may be missed in the search process; the original estimate of PA change variability (e.g. standard error) of each study should be preferable to the meta-regression analyses, rather than the used estimate based on the sample size	Interventions that attempt to attenuate the PA decline could be effective; evidence from developing and undeveloped countries is warranted; to improve the validity and comparability of instruments across studies and standardize PA definition in terms of light, moderate and vigorous intensity; aggregation of self-reported and objective PA measures
Koeneman et al. (SLR) [32]	Overall PA, overall ex, overall PA/ex	Sex, age, ethnicity, chronic conditions/ disease, general physical health, BMI	To systematically review determinants of PA and exercise among healthy older adults	The heterogeneity of the studies allowed only moderate conclusions	N.A.	There may be possibility of publication bias; a wide age range is applied that might have masked some of the differences between subsamples inside that population; they excluded some specific subsamples of the older population; overall low quality of the studies included	The determinants of PA need further study that include the use of objective measures of PA and exercise and valid and reliable measures of determinants
Siddiqi et al. (SLR) [27]	Overall PA	Physical disability/disease, fatigue, body shape/physical appearance	To systematically review the qualitative literature pertaining to impediments and enablers of PA participation among African Americans	Primary biological determinants influencing PA were fatigue and preexisting chronic diseases	N.A.	Possibility of publication bias; many included studies included only women	To effectively promote PA among African Americans, targeted interventions will need to addressimpediments at multiple levels
Andersen et al. (MA) [16]	Active vs. inactive (various definitions according studies)	Birth weight	To assess the association between birth weight and LTPA	The association between birth weight and undertaking LTPA is very weak within the normal birth weight range, but both low and high birth weights are associated with a lower probability of undertaking LTPA	OR (95%) CI for: 1.26–1.75 (kg); 1.76–2.25 (kg); 2.26–2.75 (kg); 4.26–4.75 (kg); 4.76–5.25 (kg) respectively 0.67 [0.47, 0.94]; 0.72 [0.59, 0.88]; 0.89 [0.79, 0.99]; 0.92 [0.81, 1.03]; 0.65 [0.50, 0.86] OR (95%) CI for: 1.26–1.75 (kg); 1.76–2.25 (kg); 2.26–2.75 (kg); 4.26–4.75 (kg); 4.76–5.25 (kg) respectively 0.67 [0.47, 0.94]; 0.72 [0.59, 0.88]; 0.89 [0.79, 0.99]; 0.92 [0.81, 1.03]; 0.65 [0.50, 0.86]	Some information on birth weight and all information on physical activity was self-reported	If PA constitutes a link between birth weight and morbidity and mortality, promotion of PA may be of special importance among subjects of low and high birth weights
Hinkley et al. (SLR) [21]	Overall PA	Age, sex, family risk (CVD), preterm birth, wheezing/asthma, ethnicity, BMI/weight, movement skills	To study the correlates of pre-school children’s PA behaviors	Boys were significantly more active than girls; age and BMI showed no association with PA	N.A.	Small sample sizes in included studies, as well as small variability in PA; measurement methods may not be sensitive enough; MA is impossible given the variety of effect-sizes	Simultaneous investigation of multiple variables across multiple domains may assist in the identification of potential mediating, moderating, or confounding influences on pre-school children’s PA; the use of larger samples may allow for the detection of small yet significant associations
Tzormpatzakis et al. (SLR) [23]	Total PA, leisure time PA, occupational PA, exercise and sports, exercise	Sex, age	To evaluate the evidence from research relevant to participation in PA and exercise in Greece	Men exercise more vigorously and more actively than women	N.A.	None of the studies used objective measurements and also they used different self-reported estimates; lack of appropriate use of the terms “exercise” and “PA”	PA promotion should be organized in a systematic way; intervention studies and longitudinal designs to evaluate the long-term effects are suggested; a clear definition of variables is needed; studies should concentrate on the total PA profile of the participants
Van der Horst et al. (SLR) [20]	Overall PA	Age, sex, ethnicity BMI/skinfolds	To summarize and update the literature on correlates of PA, insufficient PA, and sedentary behavior in young people	Of all potential biological determinants, sex (being male) was positively associated with PA; in children, ethnicity was found to have no effect, for adolescents some of the studies concluded that it was negatively associated with the ethnic minorities, but a final conclusion cannot be made; BMI was found to have no association in both groups	N.A.	Publication bias may be present; possibility of missed studies as a result of the search strategy; the main outcome was overall PA without other classifications; mostly cross-sectional studies included; because of the variability, it was not possible to assess the overall strength of the associations	More prospective studies are needed and more research including children
Coble et al. (SLR) [25]	Overall PA	Age, sex, body weight, health status	To understand PA behavior of Native Americans	PA levels tend to decrease with age; Native American men are more active than their female counterparts; overall, PA levels of Native Americans tend to be lower than in nonminorities; body weight showed inconsistent results	N.A.	Not all measurement methods used in the studies have been validated; only published papers were included	More studies, especially with longitudinal design are required; there is a need for application of psychological models to understand the PA motivations, as well as culturally appropriate and validated measurement tools
Rhodes et al. (MA) [26]	Overall PA	Sex, age	To understand the association between major personality traits and PA	The data for the age and sex were inconclusive, given the small number of studies, still the results of those studies suggest that these factors don’t influence personality and PA relationships	N.A.	Research is too limited to draw definitive conclusions about sex, age and culture interactions with personality and physical activity, but preliminary research suggests relative invariance	Future research using multivariate analyses, personality-channeled PA interventions, longitudinal designs and objective PA measurement is recommended

*BMI* body mass index, *CVD* cardiovascular disease, *ERS* exercise referral schemes, *MA* meta-analysis, *SLR* systematic literature review, *MVPA* moderate to vigorous physical activity, *LTPA* leisure time physical activity, *OR* odds ratio, *CI* confidence interval, *PA* physical activity, *SES* socioeconomic status

### Measurement Methods of PA

The majority of the eligible original studies used non-objective measurement methods of PA assessment (e.g., self-reporting, attendance reports) [16, 17, 19–33]. Objective measurements of PA, assessed by either accelerometer or pedometer, were used in 87 of the eligible original studies, included in 9 of the included reviews [17, 19–21, 24, 28–30, 32]. One review did not report the exact number of the studies that used objective and non-objective measures [18].

### Evaluation of the Quality of the SLRs and MAs

The results of the quality assessment are reported in Table 4. Among the 19 included reviews, 13 were of moderate quality, 2 reviews were evaluated as weak [20, 23], and 4 as strong [15, 30, 31, 34]. From those reviews that were of moderate quality, nine [17–19, 21, 22, 25, 26, 30, 33] were scored with four points and four [24, 27–29] received a quality rating of either six or seven. The characteristics of the included studies were provided by the majority of the reviews (16 out of 19 reviews); however, only 5 out of 19 reviews provided the list of the included and excluded studies. Furthermore, only 4 out of 19 reviews used the status of publication as an inclusion criterion and 2 out of 19 assessed the probability of publication bias.

**Table 4:** Quality assessment of the included reviews using the AMSTAR checklist [15]

Study	Was an “a priori” design provided?	Was there duplicate study selection and data extraction?	Was a comprehensive literature search performed?	Was the status of publication (i.e., gray literature) used as an inclusion criterion?	Was a list of studies (included and excluded) provided?	Were the characteristics of the included studies provided?	Was the scientific quality of the included studies assessed and documented?	Was the scientific quality of the included studies used appropriately in formulating conclusions?	Were the methods used to combine the findings of studies appropriate?	Was the likelihood of publication bias assessed?	Was the conflict of interest included?	Sum quality score^a^ (/11)	Quality of the review^b^
Barnett et al. [34]	Yes	Yes	Yes	No	No	Yes	Yes	Yes	Yes	Yes	Yes	9	Strong
Oglund et al. [31]	Yes	Yes	Yes	Yes	No	Yes	Yes	Yes	Yes	No	No	8	Strong
Olsen et al. [22]	Yes	No	Yes	No	No	Yes	Yes	No	N.A.	No	C.A.	4	Moderate
Babakus et al. [24]	No	Yes	Yes	Yes	No	Yes	Yes	No	Yes	No	No	6	Moderate
Barnett et al. [33]	Yes	No	C.A.	Yes	No	Yes	No	No	N.A.	No	Yes	4	Moderate
De Craemer et al. [18]	Yes	Yes	Yes	No	No	No	No	N.A.	N.A.	No	Yes	4	Moderate
Ridgers et al. [17]	Yes	C.A	Yes	No	No	Yes	No	N.A.	N.A.	N.A.	Yes	4	Moderate
Stanley et al. [19]	No	Yes	No	No	No	No	Yes	Yes	N.A.	No	Yes	4	Moderate
Uijtdewillingen et al. [28]	Yes	Yes	Yes	No	No	Yes	Yes	Yes	N.A.	N.A.	Yes	7	Moderate
Craggs et al. [29]	Yes	Yes	No	No	No	Yes	Yes	Yes	N.A.	No	Yes	6	Moderate
Dumith et al. [30]	No	No	Yes	No	Yes	Yes	No	N.A.	Yes	No	No	4	Moderate
Koeneman et al. [32]	No	Yes	Yes	No	Yes	Yes	Yes	Yes	C.A.	Yes	Yes	8	Strong
Siddiqi et al. [27]	Yes	No	Yes	No	No	Yes	Yes	Yes	N.A.	No	Yes	6	Moderate
Andersen et al. [16]	Yes	N.A.	Yes	Yes	Yes	Yes	No	No	Yes	Yes	Yes	8	Strong
Hinkley et al. [21]	Yes	Yes	Yes	N.A.	No	No	No	No	N.A.	No	Yes	4	Moderate
Tzormpatzakis et al. [23]	No	C.A	Yes	No	No	Yes	No	C.A.	N.A.	No	No	2	Weak
Van der Horst et al. [20]	No	Yes	Yes	No	No	Yes	No	N.A.	N.A.	No	No	3	Weak
Coble et al. [25]	No	No	Yes	Yes	No	Yes	No	No	Yes	No	No	4	Moderate
Rhodes et al. [26]	No	No	Yes	No	Yes	Yes	No	N.A.	No	No	Yes	4	Moderate

*C*.*A*. cannot answer, *N*.*A*. not applicable

^a^0 when the criteria were not applicable for the included review; 1 when the criteria were applicable for the included review

^b^Weak (score ranging from 0 to 3); moderate (score ranging from 4 to 7); strong (score ranging from 8 to 11) [15]

### Summary of the Results of the Included Reviews by Importance of Determinants and Strength of Evidence

Table 5 summarizes the results of the associations between the investigated biological determinants and PA, stratified in different age groups.

**Table 5:** Summary of the results of the included reviews: the importance of a determinant and its strength of evidence

Determinant	Children and adolescents	Pre-school children (overall PA)	Pre-school children (MVPA)	Children	Adolescents	Adults > 40 (overall PA)	Adults > 40 (overall ex)	Adults > 40 (overall ex/PA)	Adults < 40	All ages (≥ 18)	Rural women
Age	0, Ls [16, 19]	0, Ls [17, 22]	0, Ls [17]	0, Ls [20, 28, 29]	+, Pe [21, 28–30]	++, Pe [31]	−, Ls [31]	−, Pe [31]		++, Pe [23, 26, 27]	+, Ls [23]
Sex	+, Ls [16, 19]	0, Ls [17, 22]	+, Pe [17]	+, Pe [20, 21, 28, 29]	+, Pe [21, 29]	+, Pe [18, 31]				+, Pe [24–27]	
Ethnicity	0, Lns [16]	0, Ls [17, 22]	0, Ls [17]	0, Ls [20, 21, 29]	0, Ls [21, 28, 29]	0, Ls [31]	−, Ls [31]			++, Pe [25]	
Family risk		+, Ls [17, 22]									
Maturation	0, Ls [16, 29]			0, Ls [29]							
Special educational needs	0, Ls [16]										
Actual BMI	0, Ls [16, 19]	−, Pe [22]		0, Lns [20, 21]	0, Ls [21, 28]			+, Ls [31]		0, Ls [23, 26]	++, Ls [23]
Health status		+, Ls [22]				++, Pe [18]	+, Ls [31]	−−, Pe [31]	++, Pe [18]	++, Pe [18, 23, 26]	++, Pe [23]
Physical fitness levels (strength, endurance, coordination, agility, flexibility)	+, Lns [29]	0, Ls [17, 22]	+, Pe [17]	0, Lns [20]		++, Ls [18]			++, Pe [18]	++, Pe [18, 23]	+, Pe [23]
Birth weight	−−, Ce [17, 34]	−, Pe [17]	0, Ls [17]							+, Ce [15]	
Motor development	0, Ls [34]										
Early growth	0, Ls [34]										
Anthropometry/body shape	0, Lns [29]	−, Ls [17]		0, Ls [29]	−, Pe [29]						
Preterm birth		+, Ls [17, 22]									

*Ce* convincing evidence; *Lns* limited, no conclusive evidence; *Ls* limited, suggestive evidence; *Pe* probable evidence; *BMI* body mass index; *Ex* exercise; *PA* physical activity; *MVPA* moderate to vigorous physical activity; −− all reviews report no association between the determinant and the outcome; − association found in less than 25% of the reviews or of the original studies; 0 the variable has been found to be a determinant and/or reported a (non)-significant effect size larger than 0.30 in 25% to 75% of the available reviews or of the primary studies analyzed in these reviews; + association found in more than 75% of the reviews or of the included individual studies; ++ association found in all reviews

### Pre-School and Older Children

Among pre-school children and older children, for most of the determinants, the reviews reported mixed findings (0, (importance of determinant), Ls (strength of evidence), Table 5). However, among pre-school children, family risk, preterm birth [18, 21], and low health status [21] were negatively correlated to overall PA and/or reported an effect size larger than 0.30 in more than 75% of the identified reviews assessing these two categories of determinants (+, Ls, Table 5). The results were based on studies that were mainly cross-sectional in design. Similarly, being female [18] and lower physical fitness levels [18] are negatively related to moderate vigorous PA (MVPA) among pre-school children. These findings are based on studies of both cross-sectional and cohort study design showing fairly consistent associations (+, Pe, Table 5). BMI [21], birth weight [18] based on probable evidence (coded as (−, Pe) in Table 5), and anthropometry/body shape [18], based on limited, suggestive evidence (−, Ls, Table 5) were found to have no association with overall PA in pre-school children.

### Adolescents

In the adolescents group, increasing age and females [20, 28–30] were found to be negatively associated with PA. Because of the mixed and contradictory results in part of the studies, these associations are probable (+, Pe, Table 5*).* No association between body shape and PA among adolescents (−, Pe, Table 5) was found in one review [29].

### Children and Adolescents

Among the reviews that included children and adolescents together [17, 20, 28, 29, 34], age was found to be associated with PA (0, Ls), while sex was associated with PA (+, Ls). Birth weight [18, 31] was found not to be associated with PA with convincing strength of evidence (−−, Ce) (Table 5).

### Adults

Rural women were a particular adult category investigated by one review only [22]. It emerged that among these women, increasing age and BMI with limited, suggestive levels of evidence (+, Ls, and ++, Ls, Table 5), and lower health status and physical fitness levels with a probable level of evidence (++, Pe, and +, Pe, Table 5) respectively are negatively associated to PA.

When adults aged over 18 years were considered together, normal birth was found with convincing strength of evidence to be positively associated to PA and/or reported a significant effect size larger than 0.30 in all identified eligible studies included in the sole review assessing this particular category [16] (+, Ce, Table 5). Additionally, younger age [22, 23, 25], Caucasian ethnicity [24], better health status [22, 27, 35], and higher physical fitness levels [22, 27] were again found to be consistently positively associated to PA with a probable level of evidence among adults over 18 years of age (++, Pe, Table 5), and males [23–26] were found to be positively associated to PA in more than 75% of the included studies in the reviews (+, Pe, Table 5).

## Discussion

The aim of this umbrella systematic review was to summarize the evidence that has been produced to date about the biological determinants of PA across the life course. For most of the determinants, the strength of the level of evidence of the association with PA was mixed or probable. Few of the investigated determinants had convincing strength of evidence (Ce), either because of the lack of consistency of the results between the included studies or because of the small number of cohort studies investigating the specific determinants.

Determinants that were studied most frequently among all ages and demonstrated evidence suggesting a positive association to PA were younger age, being male, higher health status, and higher physical fitness levels.

Being female was negatively associated to PA participation in children, adolescents, and adults. The included reviews suggest that starting from adolescence and later, in adult life, increasing age is negatively associated to PA. Many reasons may explain these trends and greater understanding of the influence of additional contextual factors is required for both the sex and age determinants.

Apart from a biological background that could explain the avoidance of PA among older adults because of reduced physical capacity for everyday activities [32], other factors that change with age, such as social or behavioral, family, work status, or lifestyle, may have influence at different periods of life [36]. The observed sex difference in PA participation also may have a socio-cultural background. It is hypothesized that in women and adolescent girls, discouraging family/social environments could determine the observed sex-related differences in PA participation [37, 38]. Our findings are in line with the most recent survey on PA in the citizens of the European Union [36], which indicates steady decrease in PA participation advancing after 24 years of age and lower PA levels in females.

Among pre-school children and older children, the results were mixed, with exception of the negative association between being female and MVPA among pre-school children. The reasons behind these mixed results, as reported by the reviews, are small sample sizes, high diversity of the population included between studies, and the diversity of the measurement methods of PA used among the primary studies [18, 21].

Lower physical fitness levels and health status among adults were consistently found to be negatively associated to PA and reported as barriers to participation in PA [22, 25, 27, 32]. In contrary, PA is considered to have an important role in maintaining and improving the health status [39] indicating that special programmes targeting this particular group could be beneficial.

Normal birth weight was the only determinant for which there was convincing strength of evidence of positive association with PA among adults. This evidence is based on one MA of cohort studies that included adolescents and adults [16]. However, these results should be interpreted with caution because the quality of the individual studies included in this MA was not assessed and it included only population from the Nordic countries in Europe. Contrary to this review, two other MA and SLR that investigated the association of birth weight and PA among children and adolescents [18, 31] found no association. Although Andersen et al. [16] included adolescents in their study, they did not analyze the data in a way to assess the association specifically for this age group. However, the age stratification between younger and older than 35 years showed lower association between birth weight and PA in the younger participants’ group [16]. According to the above-mentioned reviews [16, 18, 31], normal birth weight was positively associated to PA only among adults. It is proposed that the rapid infant growth among those with lower birth weight may lead to adiposity later in life, which has negative impact on PA [31]. Based on these three reviews, it can be proposed that the normal birth weight might be positively correlated to PA among adults only.

Ethnicity was commonly studied as a determinant but, except for the adults > 18 years, the results were usually mixed or insufficient to make final conclusions. The investigated ethnic groups differ among studies and reviews, which may contribute to the inconsistency of results. Also, since many reviews compared immigrants and ethnic minorities with the general population of the countries [17–21, 24, 28, 29, 32, 33, 40], there is a possibility of bias by socioeconomic status that was not controlled in all of the individual studies.

BMI was another determinant with insufficient evidence among all age groups, due to mixed results among studies or lack of studies of longitudinal design that considered this determinant. A recent cohort study of older children showed that increased adiposity is associated to reduction of PA [41], but as yet no SLR/MA confirmed that.

Family risk for obesity and cardiovascular diseases was found to be negatively associated with PA among pre-school children, but the strength of evidence is insufficient [18, 21]. The same strength of evidence was found for most of the determinants investigated among children and adolescents, due to the large variation in the determinants investigated in different studies, which meant few could be compared, and the abundance of cross-sectional studies and lack of longitudinal investigations.

The majority of the studies included in the reviews were done in continents that include more developed countries. As a consequence, some determinants that may be characteristic and more relevant among less developed countries may not be shown.

Additionally, most of the included reviews were of moderate methodological quality. Most of them did not include gray literature and the probability of publication bias was rarely assessed. Additionally, half of the reviews did not assess the methodological quality of the studies and did not provide a list of excluded studies.

Additionally, PA was almost always assessed only in general terms (overall PA), rather than specific types of activity (e.g., leisure time, house activity, active travel) and was not defined clearly and uniformly among studies [16, 17, 20, 22–26, 29, 30, 32–34]. PA may have different patterns among sex, age, or socio-cultural contexts, which creates the possibility of bias when comparing the amount of PA between populations. Also, the lack of unified measurement methods of PA is an additional problem that was encountered among all the reviews. Specific definitions of PA may reveal greater insights into the determinants of PA behavior and together with a standardization of the assessment methods would enable a greater comparability among studies.

In addition, future studies on the mechanisms that underlie the proposed associations are needed in order to improve the knowledge about the biological determinants that influence PA.

## Conclusions

Despite the limitations, there are still recommendations that can be drawn from this umbrella review. Age, sex, birth weight, health status, and physical fitness levels should be taken into consideration when introducing interventions aimed at increasing PA. Age, sex, and birth weight are non-modifiable factors, but special attention should be given to the possible social and behavioral interactions that may cause the observed associations. Creating programmes targeted at diverse ages, female population, and people with non-normal birth weight can be helpful. In addition, since poor health status and lower physical fitness levels were often found as a barrier to participating in PA, it is recommended to adopt separate interventions according to the individual’s capacity for PA.

## Abbreviations

BMI: Body mass index
Ce: Convincing evidence
DEDIPAC: Determinants of Diet and Physical Activity
KH: Knowledge Hub
Lns: Limited, no conclusive evidence
Ls: Limited suggestive evidence
MAs: Meta-analyses
NCDs: Non-communicable diseases
PA: Physical activity
Pe: Probable evidence
SLRs: Systematic literature reviews
WHO: World Health Organization
