# Correction to: Biological determinants of physical activity across the life course: a “Determinants of Diet and Physical Activity” (DEDIPAC) umbrella systematic literature review

**DOI:** 10.1186/s40798-020-00291-6

**Published:** 2020-12-21

**Authors:** Katina Aleksovska, Anna Puggina, Luca Giraldi, Christoph Buck, Con Burns, Greet Cardon, Angela Carlin, Simon Chantal, Donatella Ciarapica, Marco Colotto, Giancarlo Condello, Tara Coppinger, Cristina Cortis, Sara D’Haese, Marieke De Craemer, Andrea Di Blasio, Sylvia Hansen, Licia Iacoviello, Johann Issartel, Pascal Izzicupo, Lina Jaeschke, Martina Kanning, Aileen Kennedy, Fiona Ling, Agnes Luzak, Giorgio Napolitano, Julie-Anne Nazare, Camille Perchoux, Tobias Pischon, Angela Polito, Alessandra Sannella, Holger Schulz, Rhoda Sohun, Astrid Steinbrecher, Wolfgang Schlicht, Walter Ricciardi, Ciaran MacDonncha, Laura Capranica, Stefania Boccia

**Affiliations:** 1 Università Cattolica del Sacro Cuore, Sezione di Igiene, Istituto di Sanità Pubblica, Rome, Italy; 2 Leibniz Institute for Prevention Research and Epidemiology-BIPS, Bremen, Germany; 3 Department of Sport, Leisure and Childhood Studies, Cork Institute of Technology, Cork, Munster Ireland; 4 Department of Movement and Sports Sciences, Ghent University, Ghent, Belgium; 5 Department of Physical Education and Sport Sciences, University of Limerick, Limerick, Ireland; 6 Department of Applied Sciences in Physical Activity and Management, Catholic University of Valencia “San Vicente Mártir”, Valencia, Spain; 7 Council for Agricultural Research and Economics, Research Centre for Food and Nutrition, Rome, Italy; 8 Department of Movement, Human and Health Sciences, University of Rome Foro Italico, Rome, Italy; 9 Department of Human Sciences, Society, and Health, University of Cassino and Lazio Meridionale, Cassino, Italy; 10 Department of Medicine and Aging Sciences, G. d’Annunzio’ University of Chieti-Pescara, Chieti and Pescara, Italy; 11 Department of Sport and Exercise Sciences, University of Stuttgart, Stuttgart, Germany; 12 Department of Epidemiology and Prevention, IRCCS Istituto Neurologico Mediterraneo: NEUROMED, Pozzilli, Italy; 13 School of Health and Human Performance, Multisensory Motor Learning Lab, Dublin City University, Dublin, Ireland; 14 Molecular Epidemiology Group, Max Delbruck Center for Molecular Medicine in the Helmholtz Association (MDC), Berlin, Germany; 15 Centre for Preventive Medicine, School of Health and Human Performance, Dublin City University, Dublin, Ireland; 16 Institute of Sport, Exercise & Active Living, Victoria University, Melbourne, Australia; 17 Institute of Epidemiology I, Helmholtz Zentrum München, German Research Center for Environmental Health, Neuherberg, Germany; 18 Centre de Recherche en Nutrition Humaine Rhône-Alpes, CarMeN INSERM U1060, University of Lyon1, Lyon, France; 19 Italian National Institute of Health, (Istituto Superiore di Sanita - ISS), Rome, Italy; 20 Fondazione Policlinico Universitario A. Gemelli IRCCS, UOC Igiene Ospedaliera, Roma, Italia

**Correction to: Sports Med Open 5, 2 (2019)**

**https://doi.org/10.1186/s40798-018-0173-9**

The original [1] omits a funding source which the authors acknowledge ahead:

Università Cattolica del Sacro Cuore contributed to the funding of this research project and its publication.
